# An adolescent rat model of vincristine-induced peripheral neuropathy

**DOI:** 10.1016/j.ynpai.2021.100077

**Published:** 2021-11-11

**Authors:** Ai-Ling Li, Jonathon D. Crystal, Yvonne Y. Lai, Tammy J. Sajdyk, Jamie L. Renbarger, Andrea G. Hohmann

**Affiliations:** a Psychological and Brain Sciences, Indiana University, Bloomington, IN, USA; b Gill Center for Biomolecular Science, Indiana University, Bloomington, IN, USA; c Program in Neuroscience, Indiana University, Bloomington, IN, USA; d Department of Pediatrics, School of Medicine, Indiana University, Indianapolis, IN, USA

**Keywords:** Chemotherapy-induced peripheral neuropathy, Adolescence, Exercise, Obesity, Neuropathic pain

## Abstract

Childhood acute lymphoblastic leukemia (ALL) is a significant clinical problem that can be effectively treated with vincristine, a vinca alkaloid-based chemotherapeutic agent. However, nearly all children receiving vincristine treatment develop vincristine-induced peripheral neuropathy (VIPN). The impact of adolescent vincristine treatment across the lifespan remains poorly understood. We, consequently, developed an adolescent rodent model of VIPN which can be utilized to study possible long term consequences of vincristine treatment in the developing rat. We also evaluated the therapeutic efficacy of voluntary exercise and potential impact of obesity as a genetic risk factor in this model on the development and maintenance of VIPN. Out of all the dosing regimens we evaluated, the most potent VIPN was produced by fifteen consecutive daily intraperitoneal (i.p.) vincristine injections at 100 µg/kg/day, throughout the critical period of adolescence from postnatal day 35 to 49. With this treatment, vincristine-treated animals developed hypersensitivity to mechanical and cold stimulation of the plantar hind paw surface, which outlasted the period of vincristine treatment and resolved within two weeks following the cessation of vincristine injection. By contrast, impairment in grip strength gain was delayed by vincristine treatment, emerging shortly following the termination of vincristine dosing, and persisted into early adulthood without diminishing. Interestingly, voluntary wheel running exercise prevented the development of vincristine-induced hypersensitivities to mechanical and cold stimulation. However, Zucker fa/fa obese animals did not exhibit higher risk of developing VIPN compared to lean rats. Our studies identify sensory and motor impairments produced by vincristine in adolescent animals and support the therapeutic efficacy of voluntary exercise for suppressing VIPN in developing rats.

## Highlights

• Vincristine treatment in adolescent rat induces significant mechanical and cold allodynia and muscle weakness.
• Voluntary exercise prevents vincristine-induced peripheral neuropathy.
• Vincristine treatment during early adolescence produces more severe peripheral neuropathy than treatment during late adolescence.
• Peripheral neuropathy induced by vincristine during adolescence persists into early adulthood.

## Introduction

Vincristine, a vinca alkaloid, is part of chemotherapeutic regimen to treat childhood leukemia and lymphoma. The current treatment has dramatically increased the survival of children with acute lymphoblastic leukemia (ALL) and improved the overall 5-year survival rate to over 90% (Hunger et al., 2012). However, nearly 80% of ALL children undergoing vincristine treatment (Lavoie Smith et al., 2015) and almost all childhood ALL survivors who were exposed to vincristine (Ramchandren et al., 2009) experience peripheral neuropathy. Such neurotoxicity is a major dosing limiting factor in vincristine treatment that prevents dose escalation for maximal disease response (Moore and Pinkerton, 2009).The long-term impact of vincristine-induced toxicity during treatment in adolescence is unknown.

A better understanding of vincristine-induced peripheral neuropathy (VIPN) and risk factors that may impact its severity and pervasiveness may facilitate efforts to prevent and treat VIPN. Such knowledge may facilitate development of effective cancer treatment with fewer unwanted side effects that also increased quality of life for the survivor. To our knowledge, only one published study has examined the severity of VIPN in developing rats (Schappacher et al., 2017). In this study, rats were exposed to vincristine during the early postnatal period (P11-P21). VIPN depends on the time of administration, the cumulative total dose and duration of the treatment (Dougherty et al., 2007, Windebank and Grisold, 2008). Clinically, older children undergoing vincristine treatment exhibited more severe VIPN compared to younger patients (Lavoie Smith et al., 2015). Therefore, it is important to assess VIPN at different developmental stages respectively. In this study, we characterized VIPN in developing rats exposed to vincristine from P35 to P49, which represents adolescence in rats (McCutcheon and Marinelli, 2009). In addition, we compared VIPN in animals at the same development stage with varying treatment regimens and different cumulative doses.

Our lab previously demonstrated therapeutic effects of voluntary exercise in a model of chemotherapy-induced peripheral neuropathy induced by the taxane chemotherapeutic agent paclitaxel. We discovered that voluntary exercise attenuated the development of paclitaxel-induced neuropathic nociception and also showed that voluntary running reduced already established mechanical and cold allodynia during the maintenance phase of paclitaxel-induced peripheral neuropathy (Slivicki et al., 2019). In addition, physical exercise has been shown to be beneficial to pain both preclinically (Pitcher, 2018) and clinically (Daenen et al., 2015, Kleckner et al., 2018, Kleckner et al., 2018, Kroll, 2015). Therefore, we investigated possible therapeutic effects of voluntary exercise on both sensory and motor effects of vincristine in adolescent rats.

A clinical review revealed that obesity increased the incidence and severity of symptoms of chemotherapy-induced peripheral neuropathy (Jesse, 2019). Clinically, we have observed that obese ALL patients developed worse VIPN than healthy weight patients (Sajdyk et al., 2020). Obesity and chronic pain/pain complaints coexist (for review see (Okifuji and Hare, 2015)), including in adolescents and children (Deere et al., 2012, Smith et al., 2014). Longitudinal studies suggest that obese people may be more at risk for developing chronic pain (Haukka et al., 2012, Heuch et al., 2013, Mork et al., 2013). In the USA, over one third of adults are obese which far exceeds the world average (Hales et al., 2017). The prevalence of obesity among children and adolescents is over 17% and is still increasing (Hales et al., 2017, Sanyaolu et al., 2019). Therefore, it is important to examine whether obesity is a risk factor in VIPN in the rodent model. We used Zucker (fa/fa) rats, the best characterized rat model of genetic obesity (Kava et al., 1990), to determine whether obesity was a risk factor in adolescent VIPN. Zucker fa/fa rats show hyperplasia compared to lean littermates as early as 17 days of age (Stern and Johnson, 1977). We, therefore, also compared VIPN between genetically mutated obese Zucker fa/fa rats and their lean littermates. Our results reveal critical periods of adolescence that are sensitive to development of VIPN, and validate the therapeutic effects of voluntary exercise for abrogating VIPN in this model.

## Materials and methods

### Subjects

Timed pregnant Sprague-Dawley rats (E14-E16) were ordered from Envigo, Indianapolis, IN, US. Litters were not weaned until P21 after birth and were housed 3 per cage, except where noted. Both sexes were included. In the exercise study, all animals (i.e. those exposed to running wheels and sedentary controls) were single housed. The temperature was kept at 73 ± 2 °F and humidity was around 45%. Animals were kept at light–dark cycles of 12/12 h. Vincristine treatment and behavioral testing began around P35 after baseline testing. Zucker fa/fa obese rats and lean littermates (P34-P36) were purchased from Charles River (Pennsylvania, USA). All the experimental procedures were approved by Bloomington Institutional Animal Care and Use Committee of Indiana University and followed the guidelines for the treatment of animals of the International Association for the Study of Pain (Zimmermann, 1983) and National Institute of Health Guide for the Care and Use of Laboratory Animals. In studies employing Zucker fa/fa obese animals, it was not possible to maintain blinding of obese vs. lean condition due to visually prominent differences in body weight between the groups. Similarly, in studies evaluating voluntary exercise, the presence of the running wheel identified exercise groups. The experimenter was always fully blinded to chemotherapy status (i.e. vincristine vs. saline) in all experiments.

### Drugs and chemicals

Vincristine was purchased from Tocris and dissolved in 0.9% saline. Vincristine at various dosage (60 µg/kg or 100 µg/kg) was administered intraperitoneally (i.p.) in a volume of 1 ml/kg following varying time points.

### Vincristine dosing regimens

The exact timing of rodent adolescence is hard to determine due to considerable individual and sex differences (McCutcheon and Marinelli, 2009, Spear, 2000). P35 to P49 was chosen as adolescent manipulation window as this period was considered to be within periadolescence in rats (Schneider, 2013).To identify the vincristine dosing regimen that results in significant peripheral neuropathy at different developmental stages, we used several vincristine dosing protocols. Dosing protocols where vincristine was injected on consecutive days produced significant VIPN: 1) 100 µg/kg once daily for 8 consecutive days from P35 to P42 or from P42 to P49 (Note: age was manipulated in separate studies, each with its own control group (vincristine vs. saline)); or 2) 100 µg/kg once daily for 15 consecutive days from P35 to P49. By contrast, vincristine injections with varying doses failed to produce robust VIPN when injected on alternate days: 3) 60 µg/kg once every other day for total of 8 injections from P35 to P49; 4) 100 µg/kg once every other day for total of 8 injections from P35 to P49. Different animals were used for each vincristine dosing regimen.

### Behavioral testing protocol

All pre-manipulation baseline responses were measured before P35. Each behavioral test was carried out once every 4 days after the beginning of treatment.

**Assessment of mechanical paw withdrawal thresholds.** Mechanical paw withdrawal threshold was assessed as described previously (Deng et al., 2016, Guindon et al., 2013, Panoz-Brown et al., 2017, Rahn et al., 2014, Smith et al., 2017)**.** Each rat was placed in a transparent Plexiglass chamber on an elevated wire mesh table and allowed to habituate for minimum of 30 min prior to testing. A rigid pipette-like filament of uniform diameter in 0.8 mm connected to an electronic von Frey aneshtesiometer (IIITC Life Science Inc., Woodland Hills, CA) was slowly and steadily approached to and against vertically to the midplantar region of the hind paw. The force was increased gradually until the animal withdrew its hind paw. Each paw was tested twice with an interval of several minutes between stimulations to avoid sensitization. Mechanical paw withdrawal thresholds, measured in grams (g), at the occurrence of withdrawal were averaged across paws because vincristine, administered systemically, induced bilateral sensitivity in hind paws.

**Assessment of sensitivity to cold.** Responsiveness to cold was assessed following assessment of mechanical paw withdrawal thresholds in the same rats as described previously (Deng et al., 2016, Guindon et al., 2013, Rahn et al., 2014). A 1-ml syringe with needle removed was filled with acetone (Sigma-Aldrich). An acetone bubble was formed at the tip of syringe by gently pushing the plunger and was then applied to the plantar surface of the hind paw with care taken to avoid contacting the paw with the syringe tip and applying mechanical pressure. The presence or absence of positive responses (flinching, licking, shaking, or biting) was recorded. Each paw was tested 5 times with an interval of several minutes between stimulations to avoid sensitization. The frequency of positive response out of 10 times of stimulation (including both paws) was counted and expressed as % Response.

**Assessment of motor function.** To investigate whether the animal’s motor function was impaired by vincristine treatment, motor performance was assessed using an accelerating Rotarod (IITC Life Science) (4–40 rpm, 300-second cutoff time) (Carey et al., 2017). Rats were trained over 2 consecutive days before baseline testing on the 3rd day. Animals were then tested once every four days throughout the entire experimental timeline. The latencies to descend from the rotating drum were measured.

**Assessment of grip strength.** Muscular strength of rats was monitored throughout the experiment using a grip strength meter (Bioseb, BIO-GS3, France). Each rat was held by its tail and lowered to grab the metal grid. Animal was then pulled backwards in the horizontal plane. The force applied to the grid just before it lost its grip was recorded as the peak tension in grams (Dong et al., 2021, Ling et al., 2007).

### Experimental protocol

**Experiment 1.** We characterized VIPN induced by various treatment regimens with different vincristine doses (60 µg/kg or 100 µg/kg, i.p.) and different time courses, as follows: 1) Adolescent rats received 8 once daily consecutive vincristine injections (100 µg/kg, i.p.) from P35 to P42; 2) Adolescent rats received 8 once daily consecutive vincristine injections (100 µg/kg, i.p.) from P42 to P49; and 3) Adolescent rats received 15 once daily consecutive vincristine injections (100 µg/kg, i.p.) from P35 to P49; 4) Adolescent rats received vincristine treatment (60 µg/kg, i.p.) once every other day from P35 to P49 for total of 8 injections; 5) Adolescent rats received vincristine (100 µg/kg, i.p.) once every other day from P35 to P49 for total of 8 injections.

**Experiment 2.** We investigated whether voluntary running could effectively block the development of VIPN induced by vincristine treatment (100 µg/kg, i.p, total of 15 once daily injections from P35 to P49) during adolescence. In this experiment, all animals were single-housed, and half of the animals (randomly assigned) were given free access to the running wheel (33 cm in diameter) throughout the experiment, whereas the other half were single housed in a regular cage without access to running wheel (i.e. sedentary control).

**Experiment 3.** To determine whether genetic obesity is a risk factor for VIPN, Zucker fa/fa obese and lean rats were used in this experiment. Animals were housed 3 per cage and received 15 once daily vincristine injections from P35 to P49. Blood glucose was measured upon the completion of the experiment. Blood was drawn with 1 ml syringe by puncturing the heart of the animals anesthetized by urethane (25%, 1.5 g/kg, i.p.). Glucose level was then read by using a diabetes testing kit (AF Contour Next EZ).

Animals in all experiments were tested once every 4 days for each of the aforementioned behavioral parameters.

### Statistical analysis

Differences in behavioral parameters and body weight were evaluated using mixed analysis of variance (ANOVA) with time points as the within-subject variable and treatment as between-subject variable, followed by Bonferroni post hoc tests. Bonferroni multiple comparison tests, which use the mean square result from the overall ANOVA table, were additionally used to determine the impact of vincristine treatment on wheel revolution at selected time points. This multiple comparison tests enable us to compare preselected and limited pairs of means to obtain more statistical power and minimize chances of type II error (Motulsky, 2013). For comparisons of blood glucose levels, two way ANOVA was used with genetic strain and treatment as independent between-subject variables. Statistical analyses were performed by using SPSS 27 (IBM) and figures were generated using GraphPad Prism version 7.04. All data are presented as mean ± S.E.M.; *P* < 0.05 was considered significant. Differences between males and females were not noted, therefore, data from both sexes were pooled for behavioral analysis.

## Results

### Vincristine treatment of 8 consecutive daily injections at 100 µg/kg during early or late phase produced mechanical and cold sensitivity

We evaluated the impact of 8 consecutive daily injections of vincristine at 100 µg/kg i.p. on sensory and motor function. We used this same dosing paradigm to investigate whether a critical time window for the development of VIPN was observed; vincristine was administered in two different phases in separate studies, either from P35 to P42 (Phase I, early phase manipulation) or from P42 to P49 (Phase II, late phase manipulation).

**Fig. 1:**
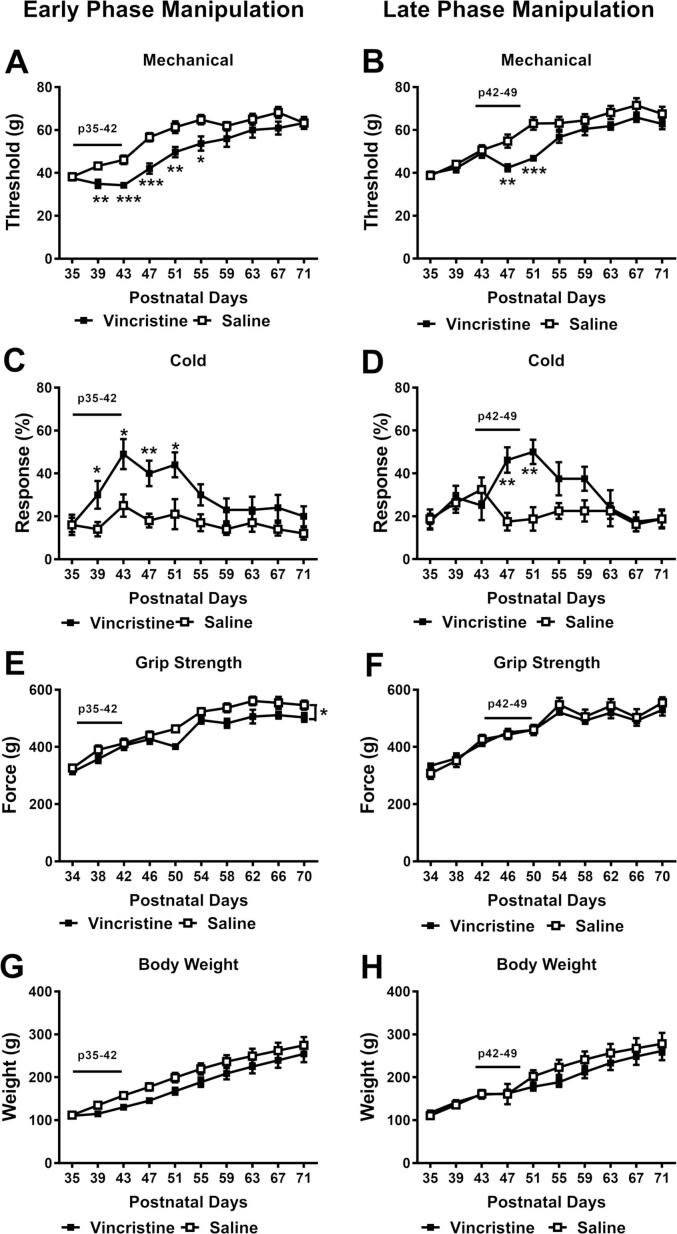
**Vincristine administration during early phase (P35-P42) produces sensory and motor neuropathy whereas vincristine administration during late phase (P42-49) produced sensory neuropathy.** Vincristine (100 µg/kg/day i.p.) for 8 consecutive days (P35-P42 or P42-P49) lowered mechanical paw withdrawal thresholds (A & B), and heightened cold sensitivity (C & D). These dosing regimens did not significantly change body weight gain (G & H). Early phase manipulation (E), but not late phase manipulation (F), impaired grip strength. Black bar indicates the timing of vincristine treatment. N = 5 males and 5 females for each group with early phase manipulation; N = 4 males and 4 females for each group with late phase manipulation. * *p* < 0.05, ** *p* < 0.01, *** *p* < 0.001 vs. saline control group.

Intensive vincristine treatment either during the early phase (Phase I; Fig. 1 A & C) or late phase (Phase II; Fig. 1 B & D) enhanced mechanical (Fig. 1 A & B) and cold hypersensitivity (Fig. 1 C & D). Mechanical paw withdrawal thresholds changed over time following Phase I (*F*_9, 162_ = 82.01, *p* < 0.01) (Fig. 1 A) or Phase II (*F*_9, 126_ = 65.67, *p* < 0.01) dosing (Fig. 1 B) with variations dependent on the chemotherapy status (Phase I: *F*_9, 162_ = 4.278, *p* < 0.01; Phase II: *F*_9, 126_ = 4.195, *p* < 0.01). Animals receiving vincristine exhibited lower mechanical paw withdrawal thresholds compared to the saline control group soon after the onset of treatment in each study (Phase I: *F*_1, 18_ = 8.333, *p* = 0.010; Phase II: *F*_1, 14_ = 5.269, *p* = 0.038). Specifically, animals receiving vincristine during Phase I (Fig. 1 A) exhibited lower mechanical thresholds on P39 to P55 and animals subjected to vincristine during Phase II (Fig. 1 B) had lower mechanical threshold on P47 to P51 compared to the saline control group. However, upon cessation of either early (Phase I) or late (Phase II) vincristine treatment, differences in the mechanical paw withdrawal thresholds between control and vincristine groups gradually disappeared.

Similarly, Phase I- vincristine treatment heightened cold sensitivity (*F*_1, 18_ = 8.319, *p* = 0.010, Fig. 1 C) whereas Phase II-treatment showed a tendency of increasing responses to cold stimulation (*F*_1, 14_ = 3.748, *p* = 0.073, Fig. 1 D). Cold hypersensitivity changed over time (Phase I: *F*_9, 162_ = 6.276, *p* < 0.01; Phase II: *F*_9, 126_ = 3.944, *p* < 0.01) and was dependent on the chemotherapy status (Phase I: *F*_9, 162_ = 1.951, *p* = 0.0483; Phase II: *F*_9, 126_ = 4.409, *p* < 0.01). Phase I- vincristine treatment induced cold hypersensitivity from P39 to P51 (Fig. 1 C); and Phase II- vincristine treatment induced cold hypersensitivity from P47 to P51 (Fig. 1 D). Cold sensitivity of vincristine-treated animals gradually attenuated over 10 days following maximal vincristine-induced cold allodynia, at which cold responsiveness did not differ from saline control group.

Vincristine treatment impaired grip strength when administered during early phase (chemotherapy status *F*_1, 18_ = 9.652, *p* = 0.006; time *F*_9, 162_ = 62.54, *p* < 0.01; interaction *F*_9, 162_ = 1.027, *p* = 0.421; Fig. 1 E), but not during late phase (chemotherapy status *F*_1, 14_ = 0.191, *p* = 0.669; time *F*_9, 126_ = 54.28, *p* < 0.01; interaction *F*_9, 126_ = 0.722, *p* = 0.688; Fig. 1 F). However, vincristine treatment during either phase failed to alter rotarod performance (Fig. S3 C&D).

Overall, these results imply that vincristine treatment during early phase had a longer lasting effect than vincristine treatment during late phase on mechanical hypersensitivity and also preferentially impacted grip strength.

By contrast, when vincristine was administered on alternate days for total of 8 injections at either 60 µg/kg or 100 µg/kg, we did not observe significant VIPN following injections of vincristine (Fig. S1, S2), although mechanical paw withdrawal thresholds were transiently reduced by vincristine at a subset of time points (Fig. S1 A & S2 A).

Animals gained weight over time (Phase I: time *F*_9, 162_ = 156.1, *p* < 0.01; Phase II: time *F*_9, 126_ = 97.01, *p* < 0.01) and body weight did not differ as a function of vincristine treatment in either early or late phase (Phase I: chemotherapy status *F*_1, 18_ = 2.349, *p* = 0.143; interaction *F*_9, 162_ = 1.082, *p* = 0.379; Phase II: chemotherapy status *F*_1, 14_ = 0.442, *p* = 0.517; interaction *F*_9, 126_ = 2, *p* = 0.044) (Fig. 1 G & H). Thus, vincristine treatment did not significantly alter normal weight gain of animals when administered during either phase.

### Long-term adolescent vincristine treatment produces mechanical and cold sensitivity and muscle weakness

**Fig. 2:**
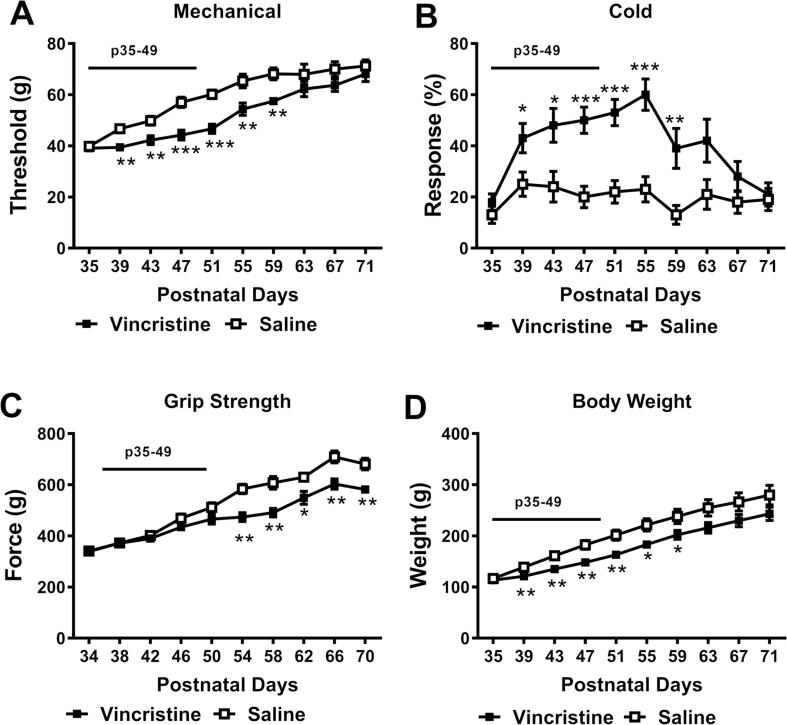
**Vincristine administration thoughout adolescence (P35-P49) produces sensory and motor neuropathy.** Vincristine (100 µg/kg/day i.p.) for 15 consecutive days (P35-P49) profoundly lowered mechanical paw withdrawal thresholds (A), heightened cold sensitivity (B), impaired the grip strength (C), and slowed body weight gain (D). Black bar indicates the period of vincristine treatment. N = 5 males and 5 females for each group. * *p* < 0.05, ** *p* < 0.01, *** *p* < 0.001 vs. saline control group.

We asked whether long-term vincristine treatment of adolescent animals would produce motor impairment (i.e. motor neuropathy) by injecting rats for 15 consecutive days with vincristine (100 µg/kg, i.p.) from P35 to P49. This vincristine regimen induced significant mechanical and cold hypersensitivity, impairment of grip strength, and also slowed weight gain (Fig. 2), but failed to alter rotarod performance (Fig. S3 E).

Both vincristine- and saline-treated groups exhibited increases in mechanical paw withdrawal thresholds as animals matured (*F*_9, 162_ = 90.86, *p* < 0.01) (Fig. 2 A). However, vincristine (100 µg/kg/day from P35-P49) slowed the normal developmental increase in mechanical paw withdrawal thresholds (*F*_9, 162_ = 3.429, *p* = 0.001) (Fig. 2 A). The vincristine-treated group displayed lower mechanical threshold compared with the saline-treated group (*F*_1, 18_ = 11.32, *p* = 0.004), specifically from P39-P59 (Fig. 2 A).

Similarly, vincristine treatment increased animals’ response to cold stimulation (*F*_1, 18_ = 20.59, *p* = 0.0003), consistent with the development of cold allodynia (Fig. 2 B). A significant main effect of time (*F*_9, 162_ = 6.716, *p* < 0.01) and significant interaction between chemotherapy status and time (*F*_9, 162_ = 3.171, *p* = 0.002) were also observed. Specifically, the vincristine-treated group exhibited greater cold sensitivity from P39-P59 (Fig. 2 B).

This same vincristine regimen (100 µg/kg/day from P35-P49) impaired the development of grip strength; vincristine treatment reduced grip strength compared to saline treatment (*F*_1, 18_ = 15.89, *p* = 0.001) (Fig. 2 C). Grip strength in both groups increased over time (*F*_9, 162_ = 115.7, *p* < 0.01) (Fig. 2 C) and the interaction between time and chemotherapy status (*F*_9, 162_ = 5.261, *p* < 0.001) was significant. Post hoc tests revealed that vincristine-treated animals exhibited weaker muscle strength compared to saline-treated counterparts from P54-P70 (Fig. 2 C), consistent with the development of vincristine-induced motor neuropathy.

Vincristine-treated animals also exhibited slower weight gain (*F*_9, 162_ = 190.1, *p* < 0.001) compared to their saline-treated counterparts (*F*_1, 18_ = 5.534, *p* = 0.030) despite both groups showing gain in body weight across the observation interval (*F*_9, 162_ = 190.1, *p* < 0.001) (Fig. 2 D). Post hoc tests revealed lower body weight in vincristine-treated animals from P39-P59.

### Vincristine treatment failed to induce VIPN in rats engaging in voluntary exercise

As the regimen of 15 once daily injections of vincristine at 100 µg/kg from P35 to P49 was most potent in inducing peripheral neuropathy in adolescent rats, we employed this vincristine regimen for all subsequent experiments.

To evaluate the effect of voluntary exercise on the development of VIPN, half of animals were allowed free access to a running wheel in the cage and the other half of animals were housed in regular cages without access to a running wheel (i.e. sedentary controls). Animals in both the exercise and sedentary groups were single housed.

**Fig. 3:**
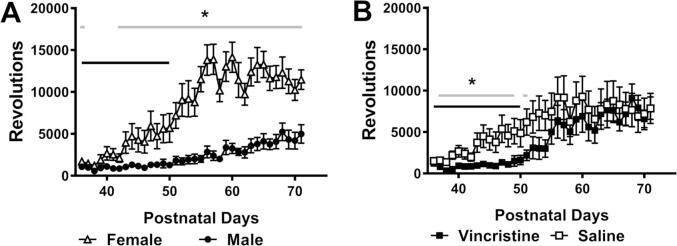
**Quantification of wheel running in the voluntary exercise groups.** (A). Overall, females exhibited significant higher running activity, defined by quantification of wheel revolutions, than males since halfway of vincristine treatment; males: N = 10, females: N = 8. (B). Vincristine treatment reduced wheel revolutions in the voluntary exercise group compared to saline treatment during the most of the treatment period (P37-P49, and P51). N = 5 males and 4 females for each group. Black bar indicates the timing of vincristine treatment; grey bar indicates the days during which significant group differences were observed. * *p* < 0.05 vs. male (A) or saline control group (B).

Overall, female rats exhibited higher levels of voluntary running activity compared to male rats, voluntary running changed across time and the interaction between sex and time was significant (sex *F*_1, 16_ = 27.584, *p* < 0.001; time *F*_35, 560_ = 29.972, *p* < 0.001; interaction *F*_35, 560_ = 10.77, *p* < 0.001) (Fig. 3 A). Despite the significant difference in the running activity between sexes, exercise prevented the reduction of mechanical paw withdrawal threshold induced by vincristine for males and females (Fig. S4). Therefore, behavioral data from both sexes were pooled to achieve higher statistical power. In addition, voluntary running activity was suppressed in vincristine-treated animals during the treatment interval as revealed by Bonferroni multiple comparison tests, although the interaction between chemotherapy status and time approached significance (chemotherapy status: *F*_1, 16_ = 1.318, *p* = 0.268; time *F*_35, 560_ = 17.351, *p* < 0.001; interaction *F*_35, 560_ = 1.398, *p* = 0.067) (Fig. 3 B).

**Fig. 4:**
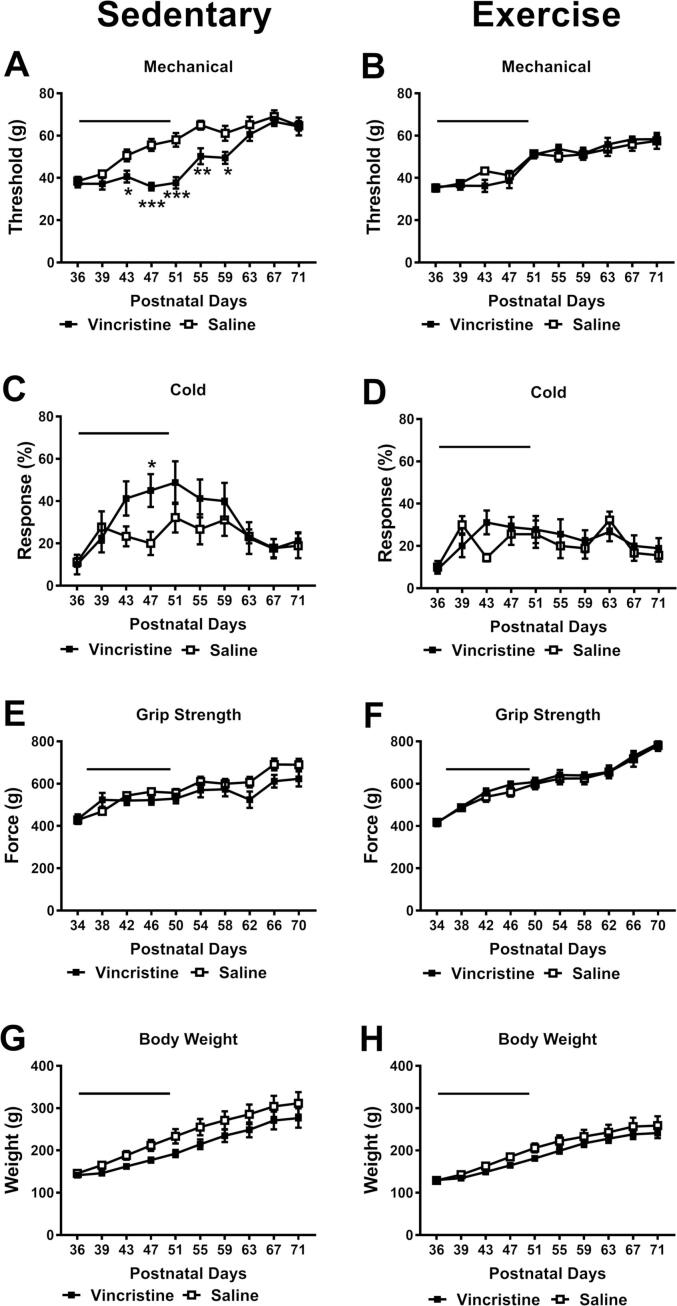
**Comparison of VIPN in sedentary and voluntary exercise conditions**. Fifteen consecutive daily injections of vincristine at 100 µg/kg/day i.p. lowered mechanical paw withdrawal thresholds in sedentary animals (A), but not in animals engaged in voluntary wheel running (B). This vincristine dosing regimen had a tendency to increase the cold sensitivty in sedentary animals (C), but did not change cold sensitivity in the exercise group (D). This vincristine treatment regimen did not impair grip strength (E & F) or slow weight gain (G & H) in either the sedentary or exercise group. Black bar indicates the period of vincristine treatment. N = 5 males and 4 females for each group; . * *p* < 0.05, ** *p* < 0.01, *** *p* < 0.001 vs. saline control group.

Both sedentary (Fig. 4 A) and exercise (Fig. 4 B) groups exhibited increased mechanical thresholds over time (sedentary: *F*_9, 135_ = 41.09, *p* < 0.001; exercise: *F*_9, 144_ = 33.07, *p* < 0.001). However, vincristine only slowed the normal developmental increase in mechanical threshold gain in sedentary (chemotherapy status: *F*_1, 15_ = 12.98, *p* = 0.0026; interaction: *F*_9, 135_ = 5.049, *p* < 0.001, Fig. 4 A) but not exercise groups (chemotherapy status: *F*_1, 16_ = 0.004, *p* = 0.953; interaction: *F*_9, 144_ = 0.988, *p* = 0.452, Fig. 4 B). Post hoc tests revealed that vincristine-treated sedentary animals exhibited lower mechanical paw withdrawal thresholds compared to saline-treated sedentary animals from P43 to P59 (Fig. 4 A). These results imply that voluntary wheel running exercise preferentially prevented the development of vincristine-induced hypersensitivity to mechanical stimulation.

Vincristine-treated sedentary animals showed increased response to cold stimuli and time dependent increases in cold responsiveness relative to saline-treated sedentary counterparts (Fig. 4 C) (time *F*_9, 135_ = 6.723, *p* < 0.001; chemotherapy status *F*_1, 15_ = 1.565, *p* = 0.230; interaction *F*_9, 135_ = 2.008, *p* = 0.043); cold responsiveness was greater in vincristine-treated sedentary rats compared to saline treated counterparts at P47 (*p* = 0.018) with a similar trend observed at P43 (*p* = 0.068). By contrast, vincristine did not alter sensitivity to cold in animals engaging in voluntary exercise (time *F*_9, 144_ = 4.294, *p* < 0.001; chemotherapy status: *F*_1, 16_ = 0.255, *p* = 0.621; interaction *F*_9, 144_ = 1.54, *p* = 0.139) (Fig. 4 D).

Grip strength in both sedentary (Fig. 4 E) (*F*_9, 135_ = 28.42, *p* < 0.001) and exercise (Fig. 4 F) (*F*_9, 144_ = 68.9, *p* < 0.001) groups increased over time. There was no main effect of chemotherapy status for either condition (sedentary: *F*_1, 15_ = 1.563, *p* = 0.230; exercise: *F*_1, 16_ = 0.370, *p* = 0.552). A significant interaction between time and chemotherapy treatment was observed for sedentary (*F*_9, 135_ = 2.601, *p* = 0.009), but not for exercise (*F*_9, 144_ = 0.182, *p* = 0.996) groups, suggesting that sedentary groups were preferentially impacted by vincristine in a time-dependent manner. Sedentary groups also trended to exhibit lower grip strength from P62 (*p* = 0.085) to P66 (*p* = 0.072) (Fig. 4 E).

Vincristine did not reliably alter body weight gain of either sedentary (Fig. 4 G) (time *F*_9, 135_ = 96.03, *p* < 0.001; chemotherapy status *F*_1, 15_ = 2.2, *p* = 0.159; interaction *F*_9, 135_ = 1.062, *p* = 0.395) or exercise (Fig. 4 H) groups (time *F*_9, 144_ = 115.7, *p* < 0.001; chemotherapy status: *F*_1, 16_ = 1.327, *p* = 0.266; interaction *F*_9, 144_ = 0.821, *p* = 0.598) throughout the observation interval.

### Obese and lean animals developed VIPN to a similar extent

To identify whether obesity is a genetic risk factor for VIPN, we evaluated VIPN in Zucker obese fa/fa animals and lean control animals. Animals were housed 3 per cage as in other experiments, with half of them receiving once daily vincristine injections (100 µg/kg from P35-P49) and the other half receiving saline as a control.

**Fig. 5:**
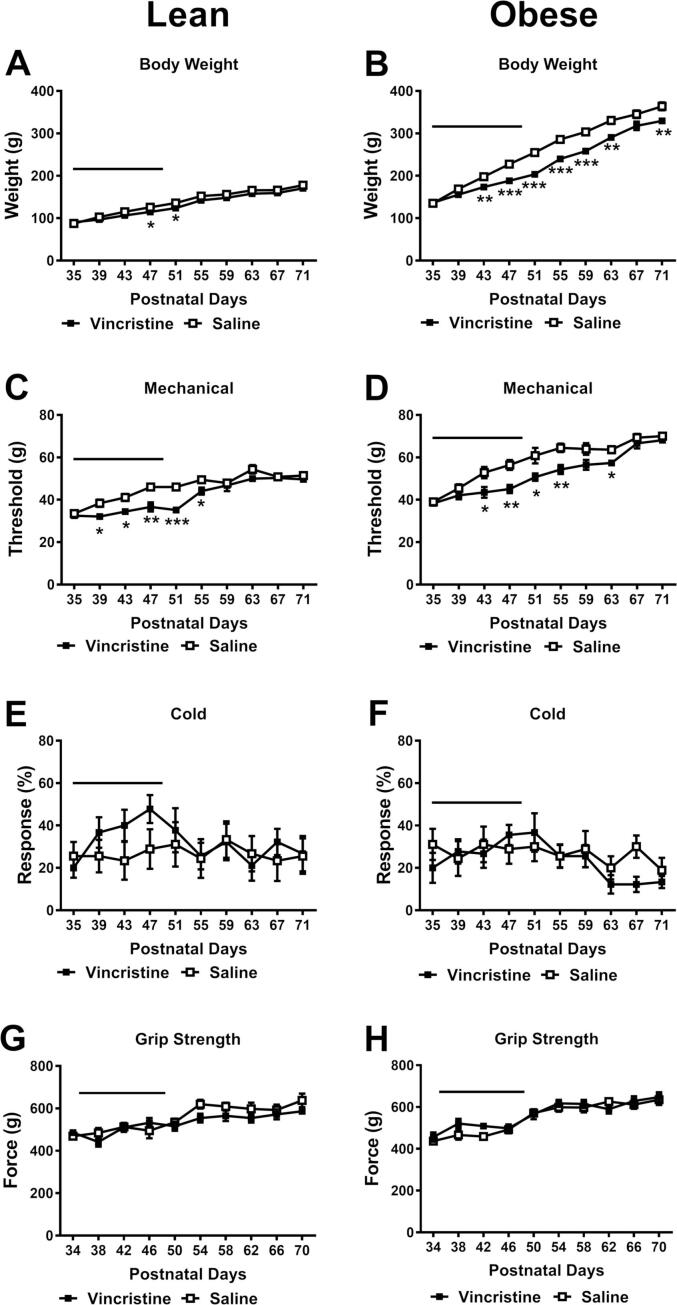
**Comparison of adolescent vincristine (P35-P49) treatment on development of VIPN in lean and Zucker obese fa/fa rats**. Fifteen consecutive once daily injections of vincristine at 100 µg/kg/day i.p. from P35-P49 lowered mechanical paw withdrawal thresholds in both lean (C) and obese (D) rats. This vincristine dosing regimen did not change cold sensitivty (E & F) or grip strength (G & H) in either lean or obese animals. Body weight gain was slowed in obese (B), but not lean (A) rats. Black bar indicates the period of vincristine treatment. N = 9 females for each group; . * *p* < 0.05, ** *p* < 0.01, *** *p* < 0.001 vs. saline control group.

Overall, obese fa/fa animals (Fig. 5B) were heavier and gained more weight than lean rats (Fig. 5 A). Vincristine treatment significantly slowed body weight gain in obese fa/fa animals and this effect was both chemotherapy status and time dependent (Fig. 5 B) (time *F*_9, 144_ = 1300, *p* < 0.001; chemotherapy status *F*_1, 16_ = 14.42, *p* = 0.002; interaction *F*_9, 144_ = 16.48, *p* < 0.001). Post hoc tests revealed that vincristine-treated obese fa/fa animals exhibited lower body weight from P43-P71. Vincristine treatment also slowed weight gain in lean rats in a time-dependent manner, but for a shorter duration, as lean rats only showed lower body weight from P47 to P51 (Fig. 5 A) (time *F*_9, 144_ = 947.9, *p* < 0.001; chemotherapy status *F*_1, 16_ = 2.764, *p* = 0.116; interaction *F*_9, 144_ = 4.82, *p* < 0.001). Glucose levels did not differ between obese and lean animals (Fig. S5).

Vincristine treatment altered mechanical paw withdrawal threshold in both obese fa/fa rats (Fig. 5 D) (time *F*_9, 144_ = 67.89, *p* < 0.001; chemotherapy status *F*_1, 16_ = 11.64, *p* = 0.004; interaction *F*_9, 144_ = 2.593, *p* = 0.008) and lean rats (Fig. 5 C) (time *F*_9, 144_ = 52.4, *p* < 0.001; chemotherapy status *F*_1, 16_ = 13.7, *p* = 0.002; interaction *F*_9, 144_ = 3.691, *p* = 0.0003). Specifically, vincristine-treated obese fa/fa animals exhibited lower mechanical withdrawal threshold from P43-P63 (Fig. 5 D) and lean animals showed lower mechanical withdrawal threshold P39-P55 (Fig. 5 C) compared to their corresponding saline-treated controls. Thus, lean rats showed an earlier onset and recovery from vincristine-induced mechanical allodynia.

Interestingly, vincristine treatment did not alter sensitivity to cold in either obese fa/fa (Fig. 5 F) (time *F*_9, 144_ = 3.077, *p* = 0.002; chemotherapy status *F*_1, 16_ = 0.276, *p* = 0.607; interaction *F*_9, 144_ = 1.34, *p* = 0.222) or lean rats (Fig. 5 E) (time *F*_9, 144_ = 2.086, *p* = 0.034; chemotherapy status *F*_1, 16_ = 0.310, *p* = 0.586; interaction *F*_9, 144_ = 1.52, *p* = 0.147), suggesting that Zucker rats may exhibit a lower propensity to develop cold allodynia compared to SD rats.

Similarly, vincristine treatment did not alter grip strength in either obese fa/fa (Fig. 5 H) (time *F*_9, 144_ = 38.75, *p* < 0.001; chemotherapy status *F*_1, 16_ = 0.652, *p* = 0.431; interaction *F*_9, 144_ = 1.315, *p* = 0.234) or lean (Fig. 5 G) (time *F*_9, 144_ = 12.77, *p* < 0.001; chemotherapy status *F*_1, 16_ = 2.026, *p* = 0.174; interaction *F*_9, 144_ = 1.251, *p* = 0.269) rats. Vincristine did not impair rotarod performance in either lean or obese animals (Fig. S6).

## Discussion

The development of VIPN is related to cumulative total dose and time of administration in adult rodents (Dougherty et al., 2007, Windebank and Grisold, 2008). Here we show that vincristine treatment during the critical developmental period of adolescence produced both sensory and motor impairments that differed based upon timing of vincristine treatments. When vincristine (100 µg/kg, i.p.) was administered once daily for 8 days consecutively during early (P35-P42) or late (P42-P49) phase, we observed lower mechanical withdrawal threshold and heightened cold sensitivity in rats following the initiation of vincristine treatment. By contrast, 8 spaced injections (i.e. on alternate days) of vincristine at either 60 µg/kg or 100 µg/kg from P35 to P49 did not produce pronounced VIPN. Strikingly, following 8 consecutive once daily injections, grip strength was impaired when vincristine was administered during the early phase, but not when the same doses were administered during the late phase. These latter observations suggest that vincristine treatment during early phase may cause more severe VIPN compared to late phase in our rodent model. As expected, 15 consecutive daily vincristine injections (100 µg/kg/day, i.p.) throughout the adolescence produced the most profound VIPN. VIPN outlasted the period of active vincristine dosing, consistent with clinical observations (Ramchandren et al., 2009). The persistence of VIPN observed here is likely to be clinically relevant, as two weeks in the lifespan of an adult rat is reported to be analogous to at least one human year (Agoston, 2017, Sengupta, 2013). Nevertheless, vincristine-induced mechanical and cold sensitivity eventually subsided over time after the cessation of vincristine treatment to levels observed in saline-treated rats. By contrast, vincristine-induced impairment of grip strength only manifested itself after the termination of vincristine treatment, and was long-lasting and persisted into adulthood without diminishing. These observations indicate that the vincristine effect had different time courses of impairment on motor and sensory function.

Vincristine-induced mechanical and cold hypersensitivity outlasted the duration of vincristine treatment and persisted into early adulthood. However, the observed mechanical and cold hypersensitivities eventually disappeared in weeks following cessation of vincristine treatment. This is in accordance with vincristine effects in adult rats where animal’s nociceptive responses were normalized during the two weeks following discontinuation of vincristine treatment (Aley et al., 1996). Clinically, very few studies investigated the long-term consequences of vincristine exposure during adolescence. Ramchandren et al. (2009) studied survivors of childhood ALL who were at least 2 years off ALL therapy and found that nearly all the childhood ALL survivors studied exhibited neuropathy. However, pain was not singled out in this study and survivors were not followed up long enough to determine whether the observed neuropathy was permanent. In our study, vincristine-treated adolescent rats developed mechanical and cold hypersensitivity within a few days following the initiation of the treatment. However, when vincristine was administered during the pre-adolescent period (i.e. P11-P21), mechanical hypersensitivity did not emerge until a few days following completion of the treatment and animal’s response to noxious heat and cold were unaffected (Schappacher et al., 2017). By contrast, heat sensitivity developed in adult rats exposed to vincristine (Aley et al., 1996), whereas other vincristine dosing protocols documented robust mechanical hypersensitivity in the absence of heat sensitivity (Rahn et al., 2007). These variations in VIPN may also be due to different timeframes when vincristine was administered. Clinically, the total neuropathy score observed in children with ALL was positively associated with age, in that older children scored higher in most total neuropathy items (Lavoie Smith et al., 2015).

We did not observe significant impairment in rotarod performance in any of our vincristine dosing regimens. Aley et al. (1996) discovered that adult rats receiving daily vincristine intravenously at 200 µg/kg, but not at 100 µg/kg, over a period of two weeks, developed severe impairment in rotarod performance. This observation implies that demonstrations of loss of motor function may require a higher vincristine dose. In our study, vincristine slowed, but did not eliminate, body weight gain across time, which is similar to what was observed when animals were exposed to vincristine at earlier time points (P11 to P 21) (Schappacher et al., 2017). By contrast, adult animals exposed to vincristine showed an absence of weight gain (Aley et al., 1996, Rahn et al., 2007, Weng et al., 2003) and regained weight upon ceasing of the treatment (Aley et al., 1996). We did not test vincristine doses higher than 100 µg/kg/day because unacceptable mortality rates were observed in younger rats receiving 60 µg/kg/day after the third consecutive dose (Schappacher et al., 2017).

Regular exercise has been increasingly recommended and prescribed for chronic pain conditions (Macfarlane et al., 2017, Qaseem et al., 2017). However, an acute bout of exercise usually resulted in unchanged or even increased pain sensitivity in chronic pain patients (Rice et al., 2019, Sluka et al., 2018). Preclinically, regular exercise was therapeutically beneficial in various models of chronic pain (for review (Pitcher, 2018)). Our lab has previously shown that voluntary exercise during the development phase of CIPN prevented paclitaxel-induced neuropathy. We also showed that voluntary running prior to paclitaxel treatment delayed the onset of neuropathic nociception whereas voluntary running during the maintenance phase of CIPN markedly reduced neuropathic nociception (Slivicki et al., 2019). Here, we investigated whether voluntary exercise would prevent the development of VIPN in adolescent mice treated with vincristine. Animals were allowed free access to running wheels throughout the experiment beginning with the initiation of vincristine treatment to enhance translational relevance. No difference was observed in mechanical and cold responsiveness observed between exercise groups receiving vincristine or saline treatments, indicating that voluntary exercise prevented the development of VIPN. The lower mechanical paw withdrawal threshold in the saline-treated exercise group compared to the saline-treated sedentary group could possibly be explained by exercise-induced alterations in endogenous analgesic systems or lower body weight in the exercise group. We did not observe vincristine-induced impairment in grip strength in either exercise or sedentary animals. The lack of grip strength impairment in sedentary animals is discordant with our previous experiment where the same vincristine regimen was administered. One possible explanation would be the different housing conditions. The sedentary animals were single housed to match the exercise animals, whereas animals were group housed in the previous experiment. Single-housed rats tend to be more stressed than group-housed rats, which may contribute to the variations in the grip strength test which is influenced by animal’s stress level. We observed that vincristine-treated rats showed lower levels of voluntary running than control animals during the treatment period and confirmed that female rats showed greater levels of voluntary running than male rats. Nevertheless, effects of vincristine measured here did not differ between female and male animals, indicating that male rats showed sufficient levels of voluntary exercise to produce a complete protective effect against the development of VIPN. Other clinical and preclinical studies have suggested that the benefit of exercise is not intensity-dependent; it has been reported that exercise benefits do not seem to be related to the type of injury or the exercise paradigm (Pitcher, 2018). Taken together, these results suggest that exercise can be an effective non-pharmacological measure to prevent VIPN.

Interestingly, Zucker obese fa/fa rats did not display higher risk of developing VIPN than their lean littermates in our study, suggesting that genetic predisposition to obesity alone (i.e. in the absence of dietary factors) may be insufficient to enhance VIPN. Preclinical research on the relationship between pain and obesity has yielded conflicting results. Some studies discovered that obesity potentiated pain behaviors (Guillemot-Legris et al., 2018, Iannitti et al., 2012, Roane and Porter, 1986, Song et al., 2018, Song et al., 2017, Tramullas et al., 2016), while other studies either found no impact of obesity on pain (Croci and Zarini, 2007, Iannitti et al., 2012) or even reduced nociceptive responses in obese animals (Ramzan et al., 1993, Rodgers et al., 2014). These variations might be due to the different obesity and pain models employed in different studies. For example, genetically mutated obese Zucker rats displayed shorter latencies in tail flick test (Roane and Porter, 1986), suggestive of an acute nociceptive hypersensitivity, while high-fat diet induced obesity increased tail flick latency, suggestive of hypoalgesia (Ramzan et al., 1993). In addition, Iannitti et al. (2012) showed that Zucker obese rats exhibited increased pain sensitivity in response to intradermal carrageenan but not to paw-incision or intraplantar injection of capsaicin. Interestingly, when both Sprague-Dawley rats and Long-Evans rats were maintained on a high-fat diet, only Long-Evans rats gained significant body weight and exhibited hallmarks of obesity (e.g. increased body fat, plasma leptin and insulin, etc.) despite the fact that both Sprague-Dawley and Long-Evans rats displayed enhanced pain sensitivity (Song et al., 2017). These findings suggest that factors other than body weight contribute to pain progression. Special diets are not needed for Zucker fa/fa rats to develop obesity as they exhibit hyperphagia and gain excessive weight on low fat or even restricted diets (Zucker and Zucker, 1962). Zucker rats in our study were on a diet of regular chow. More work is necessary to determine whether high-fat diet-induced obesity potentiates the development of VIPN in adolescent rats. Only female Zucker rats were included in this study because most of the obese patients included in our previous clinical study of ALL patients with VIPN were females (Sajdyk et al., 2020). Therefore, a possible role for sex in the impact of obesity on the VIPN remains to be explored. Song et al. (2018) showed that the effect of high-fat diet was smaller in females compared to male rats. We did not observe differences in the blood glucose level between Zucker obese and lean rats, which is in concordance with observations that Zucker fa/fa obese rats are normoglycaemic (Iannitti et al., 2012, Zucker and Zucker, 1962). This suggests that glucose level did not contribute to the presence or absence of obesity-enhanced pain sensitivity in Zucker (fa/fa) obese rats. Collectively, these studies indicate that the relationship between obesity and pain is complex and multifaceted.

In conclusion, we established an adolescent model of VIPN in which 15 consecutive daily vincristine treatment (100 µg/kg, i.p.) from P35 to P49 produced mechanical and cold allodynia, as well as weakened grip strength. Voluntary exercise was effective in fully preventing the development of VIPN. Genetically mutated Zucker obese female rats, used as a genetic model of juvenile obesity, did not show increased propensity to develop VIPN. The present observations may help guide future translational research aimed at identifying risk factors for VIPN in adolescents as well as non-pharmacological environmental interventions such as voluntary exercise that are efficacious in suppressing development of VIPN in people.

### CRediT authorship contribution statement

**Ai-Ling Li:** Investigation, Data curation, Methodology, Writing – original draft. **Jonathon D. Crystal:** Writing – review & editing, Funding acquisition. **Yvonne Y. Lai:** Writing – review & editing, Funding acquisition. **Tammy J. Sajdyk:** Writing – review & editing, Funding acquisition. **Jamie L. Renbarger:** Funding acquisition. **Andrea G. Hohmann:** Conceptualization, Methodology, Writing – review & editing, Supervision, Funding acquisition.

## Declaration of Competing Interest

The authors declare that they have no known competing financial interests or personal relationships that could have appeared to influence the work reported in this paper.
