# Supplementary material for: An adolescent rat model of vincristine-induced peripheral neuropathy
Source: Neurobiol Pain. 2021 Nov 11;10:100077. doi: 10.1016/j.ynpai.2021.100077 (PMC8605395; doi:10.1016/j.ynpai.2021.100077)
Supplement: Supplementary data 1 [file mmc1.docx]

**SUPPLEMENTAL FIGURES** **
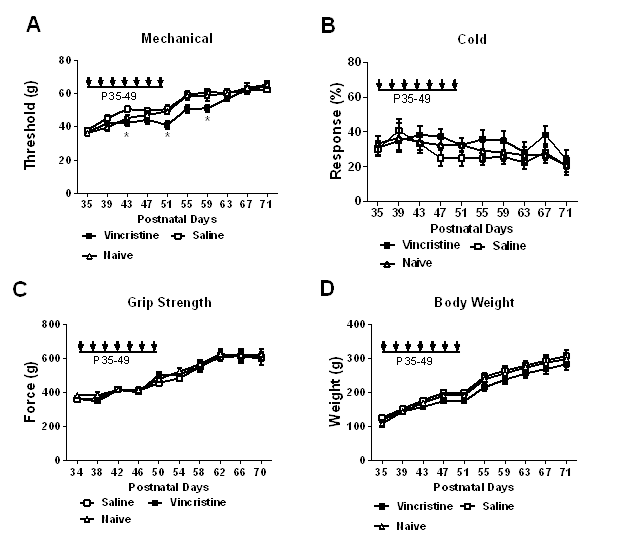
Figure S1. Vincristine injection at 60 µg/kg i.p. once every other day for total of 8 injections from P35 to P49 failed to produce pronounced VIPN.** This regimen lowered mechanical paw withdrawal threshold at P43, 51 and 59 (A); however it did not potentiate cold sensitivity (B), impair grip strength (C), or slow weight gain (D). We did not observe any differences between a naïve group and saline-injected controls. N = 8 males and 4 females for each group. Black bar indicates the vincristine treatment period and downward arrows indicate time points of injection. * *p* < 0.05 vs. saline control group.


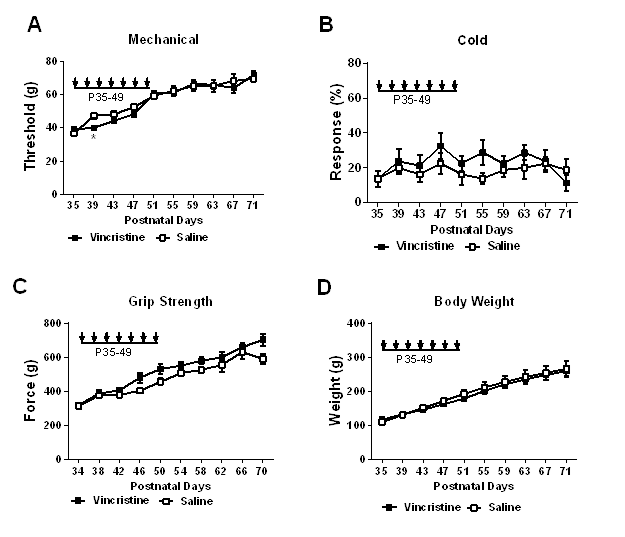


**Figure S2. Vincristine injection at 100 µg/kg i.p. once every other day for a total of 8 injections from P35 to P49 did not produce pronounced VIPN.** This regimen induced lower mechanical threshold at P39 (A); however it did not heighten cold sensitivity (B), impair grip strength (C), or slow body weight gain (D). N = 4 males and 4 females for each group. Black bar indicates the vincristine treatment period and downward arrows indicate time points of injection. * *p* < 0.05 vs. saline control group.


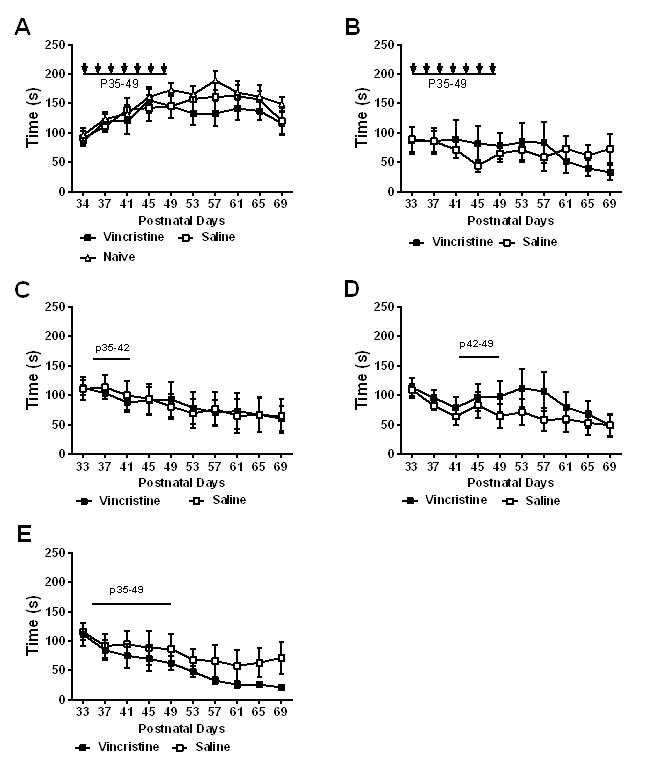


**Figure S3. Vincristine dosing regimens during adolescence did not reliably produce motor ataxia in the rota-rod test**. (A), Spaced (i.e. injection on alternate days) vincristine injections at 60 µg/kg/day i.p. for a total of 8 injections. (B), Spaced vincristine injections at 100 µg/kg i.p. for a total of 8 injections. (C), Eight consecutive daily vincristine injections (100 µg/kg/day, i.p.) during early phase from P35-42. (D), eight consecutive daily vincristine injections (100 µg/kg/day, i.p.) during late phase from P42-49. (E), fifteen consecutive daily vincristine injections (100 µg/kg/day, i.p.) throughout adolescence from P35-49. Black bar indicates the timing of vincristine treatment and downward arrows indicate time points of injection.


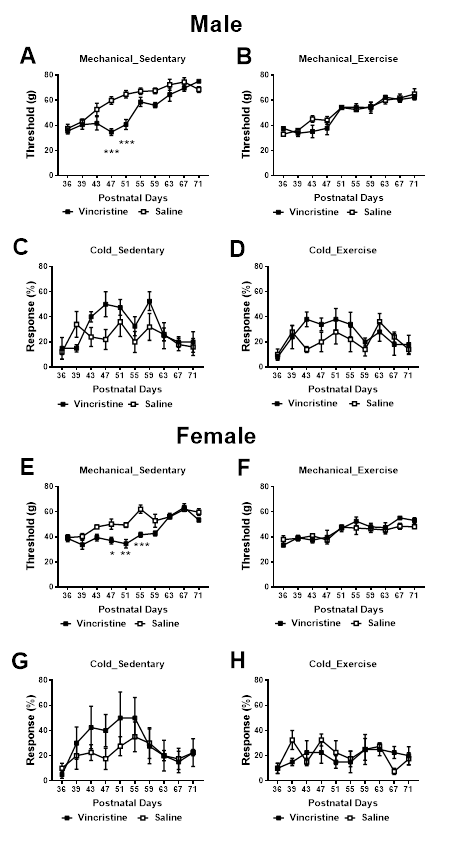


**Figure S4. Exercise prevented the development of mechanical hypersensitivity in both male and female rats.** Vincristine induced mechanical hypersensitivity in both male (A) and female (E) rats, and exercise prevented the development of mechanical hypersensitivity induced by vincristine in both male (B) and female (F) rats. Significant effect of vincristine on response to cold stimulation in sedentary animals (C & G) was not detected when groups are separated by sex. There is no difference in the cold response between saline- and vincristine-treated exercise animals (D & H).


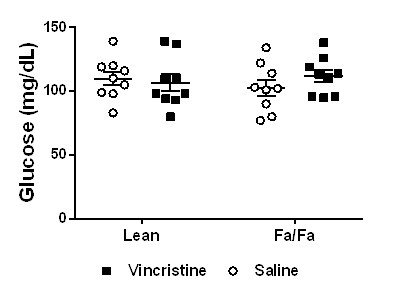


**Figure S5. Glucose levels did not differ between obese fa/fa and lean rats.** Behavioral data from these same animals is shown in Figure 5. N = 9 females per group.


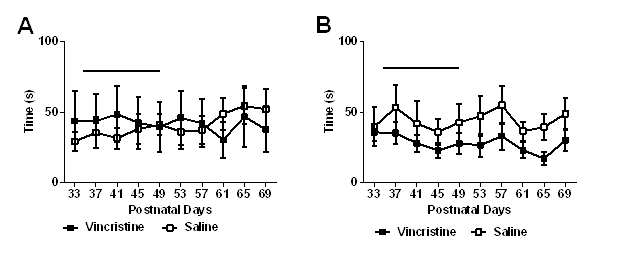


**Figure S6. Fifteen consecutive daily vincristine injections at 100 µg/kg/day i.p. from P35-P49 failed to induce motor impairment in either lean (A) or obese animals (B).** Black bar indicates the vincristine treatment period.
